# Perturbation of PALB2 function by the T413S mutation found in small cell lung cancer

**DOI:** 10.12688/wellcomeopenres.13113.2

**Published:** 2018-01-18

**Authors:** Jean-Yves Bleuyard, Rosie M. Butler, Fumiko Esashi

**Affiliations:** 1 Sir William Dunn School of Pathology, University of Oxford, Oxford, OX1 3RE, UK; 2 St John's Institute of Dermatology, Division of Genetics and Molecular Medicine, Faculty of Life Sciences & Medicine, King's College London, London, SE1 9RT, UK

**Keywords:** PALB2, small cell lung cancer, DNA damage, camptothecin

## Abstract

**Background:** Germline mutations in the *PALB2* gene are associated with the genetic disorder Fanconi anaemia and increased predisposition to cancer. Disease-associated variants are mainly protein-truncating mutations, whereas a few missense substitutions are reported to perturb its interaction with breast cancer susceptibility proteins BRCA1 and BRCA2, which play essential roles in homology-directed repair (HDR). More recently, PALB2 was shown to associate with active genes independently of BRCA1, and through this mechanism, safeguards these regions from DNA replicative stresses. However, it is unknown whether PALB2 tumour suppressor function requires its chromatin association.

**Methods:** Mining the public database of cancer mutations, we identified four potentially deleterious cancer-associated missense mutations within the PALB2 chromatin association motif (ChAM). To assess the impact of these mutations on PALB2 function, we generated cell lines expressing PALB2 variants harbouring corresponding ChAM mutations, and evaluated PALB2 chromatin association properties and the cellular resistance to camptothecin (CPT). Additionally, we examined the accumulation of γH2A.X and the RAD51 recombinase as readouts of DNA damage signalling and HDR, respectively.

**Results:** We demonstrate that a small-cell lung cancer (SCLC)-associated T413S mutation in PALB2 impairs its chromatin association and confers reduced resistance to CPT, the only FDA-approved drug for relapsed SCLC. Unexpectedly, we found a less efficient γH2A.X nuclear foci formation in PALB2 T413S expressing cells, whereas a near-normal level of RAD51 nuclear foci was visible.

**Conclusions:** These findings support the importance of PALB2 chromatin association in the suppression of tumours, including SCLC, an unusually aggressive type of cancer with poor prognosis. PALB2 T413S has little impact on RAD51 recruitment, likely due to its intact interaction with BRCA1 and BRCA2. However, this mutant shows inefficient DNA stress signalling. This finding sheds new light on the function of PALB2, playing a role in efficient DNA stress signalling through constitutive chromatin association. ****

## Introduction

*BRCA1* and *BRCA2* (breast cancer 1 and 2) are two of the best-known cancer susceptibility genes, and mutations in these genes are causally connected to the rare genetic disorder Fanconi anaemia. At the molecular level, BRCA1 and BRCA2 cooperate to promote the homology-directed repair (HDR) of highly genotoxic DNA lesions, such as double-strand breaks (DSBs) or inter-strand crosslinks (ICLs) ^1– 3^. PALB2 (Partner And Localizer of BRCA2) was more recently identified as a binding partner of BRCA2 and was subsequently shown to bridge BRCA1 and BRCA2 physically ^4– 7^. Importantly, following studies have demonstrated a link between germline mutations in the *PALB2* gene, and Fanconi anaemia and an elevated risk of developing breast, ovarian and pancreatic cancers ^8– 12^, identical to cancer types associated with *BRCA1* and *BRCA2* germline mutations ^13– 15^.

To date, the majority of disease-associated *PALB2* mutations are loss-of-function mutations caused by C-terminal protein truncations of various lengths ^10, 16, 17^. The PALB2 C-terminal WD40-type β-propeller domain is known to promote interaction with BRCA2, and its structure has demonstrated that deleting only the last four residues (the consequence of the PALB2 Y1183X cancer-associated nonsense mutation) is sufficient to disrupt the proper folding of the domain and render the protein unstable ^5, 18, 19^. Another study has revealed that cancer-associated PALB2 C-terminal truncations (such as Q988X or W1038X) can alternatively expose a hidden nuclear export signal (residues 928-945) and lead to the mis-localisation of PALB2 in the cytoplasm ^20^. Since PALB2 forms homo-oligomers through its N-terminal coiled-coil domain ^19^, it is plausible that the Q988X and W1038X truncated proteins can sequester the product of a functional *PALB2* allele in the cytoplasm. These variants are hence unable to fulfil PALB2 tumour suppressor function, even in the presence of a wild-type allele.

Recent studies have additionally characterised several missense mutations of PALB2 associated with human disease. For example, an in-frame exclusion of PALB2 exon 6, resulting in the deletion of residues 839-862 within the WD40 repeat domain, has been reported to give rise to a mild form of Fanconi anaemia, without the severe developmental abnormalities usually associated with the disease ^21^. In line with this observation, when exogenously expressed in U2OS cells, this hypomorphic in-frame exclusion variant of PALB2 retained BRCA2 interaction and supported RAD51 accumulation at damage-induced nuclear foci, a crucial step of DSB and ICL repair mediated by the BRCA1-PALB2-BRCA2 complex. Conversely, *PALB2* sequencing in non- *BRCA1/2* familial breast cancer cases led to the identification of germline missense mutations in the coiled-coil domain (L35P), identified in patients with a truncating mutation in the second allele, and WD40 repeat domain (L939W and L1143P) ^22, 23^. These single-residue changes result in decreased interaction with BRCA1 and BRCA2, respectively, and have been shown to reduce the cellular DSB-repair potential ^22, 23^. Additionally, breast cancer-associated germline mutations in BRCA2 N-terminus (G25R, W31C and W31R) have also been reported to disrupt the PALB2-BRCA2 interaction ^5^. These findings emphasise the critical role of the BRCA1-PALB2-BRCA2 complex in the maintenance of genome stability and prevention of cancer and provide evidence that heterozygosity for rare missense ** variants of PALB2 may influence cancer risk.

We previously described the evolutionarily conserved chromatin association motif (ChAM) of PALB2 and showed that it promotes direct interaction with nucleosomes ^24, 25^. Together with the MRG15-binding domain (MBD), which mediates PALB2 interaction with the chromodomain-containing MRG15 protein, ChAM controls PALB2 chromatin association ^24, 26^. In our latest study, we further demonstrated that the ChAM and the MBD act in concert to tether PALB2 to active genes, protecting these loci from replication-associated stress ^25^. Our analysis of missense substitutions within the MBD established that, in the absence of this mechanism, cells accumulate DNA damage at active genes, a process that may ultimately lead to the conversion of pre-cancerous to cancerous cells. Given the emerging evidence that missense substitutions in protein-binding domains of PALB2 are connected with disease predisposition, we aimed to elucidate whether cancer-associated missense mutations may also perturb the function of the PALB2 ChAM.

## Methods

### Sequence analyses

Sequences of PALB2 orthologues from 40 species were retrieved from the Ensembl and NCBI resources, and aligned with MUSCLE. Dr ( *Danio rerio*, Zebrafish), Tr ( *Takifugu rubripes*, Japanese pufferfish), On ( *Oreochromis niloticus*, Nile tilapia), Ac ( *Anolis carolinensis*, Carolina anole lizard), Xt ( *Xenopus tropicalis*, Western clawed frog), Sh ( *Sarcophilus harrisii*, Tasmanian devil), Md ( *Monodelphis domestica*, Gray short-tailed opossum), Sa ( *Sorex araneus*, Common shrew), Ml ( *Myotis lucifugus*, Little brown bat), Cp ( *Cavia porcellus*, Guinea pig), Ss ( *Sus scrofa*, Wild boar), La ( *Loxodonta africana*, African bush elephant), Cg ( *Cricetulus griseus*, Chinese hamster), Mm ( *Mus musculus*, House mouse), Rn ( *Rattus norvegicus*, Brown rat), Ch ( *Choloepus hoffmanni*, Two-toed sloth), Am ( *Ailuropoda melanoleuca*, Giant panda), Mpf ( *Mustela putorius furo*, Ferret), Dn ( *Dasypus novemcinctus*, Nine-banded armadillo), Sbb ( *Saimiri boliviensis boliviensis*, Bolivian squirrel monkey), Gog *(Gorilla gorilla gorilla*, Western lowland gorilla), Hs ( *Homo sapiens*, human), Cj ( *Callithrix jacchus*, Common marmoset), Pt ( *Pan troglodytes*, Chimpanzee), Nl ( *Nomascus leucogenys*, White-cheeked gibbon), Mam ( *Macaca mulatta*, Rhesus macaque), Pa ( *Pongo abelii*, Sumatran orangutan), Paa ( *Papio anubis*, Olive baboon), Clf ( *Canis lupus familiaris*, Dog), Ord ( *Odobenus rosmarus divergens*, Walrus), Bt ( *Bos taurus*, Cow), Oa ( *Ovis aries*, Sheep), Vp ( *Vicugna pacos*, Alpaca), Tt ( *Tursiops truncates*, Common bottlenose dolphin), Oo ( *Orcinus orca*, Killer whale), Tg ( *Taeniopygia guttata*, Zebra finch), Gg ( *Gallus gallus*, Red junglefowl), Mg ( *Meleagris gallopavo*, Wild turkey), Ps ( *Pelodiscus sinensis*, Chinese softshell turtle) and Cm ( *Chelonia mydas*, *Green sea turtle*).

Polyphen-2 prediction of potentially deleterious amino acid substitutions was performed using the Harvard webserver default parameters. The amino acid substitutions were submitted for batch analysis using human PALB2 Q86YC2 (Uniprot) sequence as reference.

SIFT prediction of potentially deleterious amino acid substitutions was performed using the J. Craig Venter Institute webserver default parameters. The amino acid substitutions were submitted for batch analysis using human PALB2 ENSP00000261584 (Ensembl) sequence as reference.

The secondary structure elements of the human ChAM (PALB2 residues 395 to 450) were predicted using the University of Dundee Jpred4 ^27^ webserver default parameters.

### Cell culture and cell lines

HEK293T and EUFA1341 (PALB2-deficient) cells were grown in Dulbecco’s Modified Eagle’s Medium (DMEM, D6429, Sigma-Aldrich) supplemented with 10% (v/v) FBS (foetal bovine serum), penicillin (100 U/ml) and streptomycin (0.1 mg/ml). All cells were grown at 37°C in an incubator containing 5% CO _2_. EUFA1341 cells were transfected with pCEP4-GW/FLAG-PALB2 variants plasmids. Stable cell lines expressing PALB2 cancer-associated variants were first selected and later maintained with 300 µg/ml and 150–200 µg/ml hygromycin, respectively. HEK293T (CRL-3216) were obtained from ATCC and EUFA1341 cells were a kind gift from Dr. H. Joenje (VU University Medical Center, The Netherlands).

### Plasmids

ChAM and full-length PALB2 point mutations were introduced in Gateway entry vectors (Invitrogen) using the QuikChange Site-Directed Mutagenesis Kit (Stratagene) and confirmed by DNA sequencing. Table 1 provides a list of the mutagenic oligonucleotides used in this study. For GFP-ChAM expression in HEK293T cells, ChAM variants in pENTR1A were transferred to pcDNA-DEST53 (12288015, Invitrogen) using Gateway cloning. For FLAG-PALB2 expression in EUFA1341 cells, PALB2 variants in pENTR3C were transferred to pCEP4-GW/N3xFLAG ^25^ using Gateway cloning.

**Table 1.** List of oligonucleotides used for mutagenesis.

Residue change(s)	Primer sequence (5’ → 3’); F: forward; R: reverse.
**T397S**	F: AAGGCCTTCAGGCACTGAGCAAGAATGTTTTTCTG R: CAGAAAAACATTCTTGCTCAGTGCCTGAAGGCCTT
**F404L**	F: CATAATATTCTGCAGGTAACAGAAGGCCTTCAGGC R: GCCTGAAGGCCTTCTGTTACCTGCAGAATATTATG
**P405T**	F: GCCTGAAGGCCTTCTGTTTACTGCAGAATATTATGTTAG R: CTAACATAATATTCTGCAGTAAACAGAAGGCCTTCAGGC
**F404A/P405A**	F: ACAGTGCCTGAAGGCCTTCTGGCTGCTGCAGAATATTATGTTAGAAC R: GTTCTAACATAATATTCTGCAGCAGCCAGAAGGCCTTCAGGCACTGT
**Y408H**	F: CGTGTTGTTCTAACATAATGTTCTGCAGGAAACAGAAGGC R: GCCTTCTGTTTCCTGCAGAACATTATGTTAGAACAACACG
**E407A/Y408A**	F: GAAGGCCTTCTGTTTCCTGCAGCAGCTTATGTTAGAACAACACGAAGC R: GCTTCGTGTTGTTCTAACATAAGCTGCTGCAGGAAACAGAAGGCCTTC
**V410G**	F: GTTTCCTGCAGAATATTATGGTAGAACAACACGAAGCATG R: CATGCTTCGTGTTGTTCTACCATAATATTCTGCAGGAAAC
**T413S**	F: CAGAATATTATGTTAGAACATCACGAAGCATGTCCAATTG R: CAATTGGACATGCTTCGTGATGTTCTAACATAATATTCTG
**V410P/T413P**	F: GCCTTCTGTTTCCTGCAGAATATTATCCTAGAACACCACGAAGCATGTCC R: GGACATGCTTCGTGGTGTTCTAGGATAATATTCTGCAGGAAACAGAAGGC
**S417Y**	F: GAACAACACGAAGCATGTACAATTGCCAGAGGAAAG R: CTTTCCTCTGGCAATTGTACATGCTTCGTGTTGTTC
**N418A/C419I**	F: TAGAACAACACGAAGCATGTCCGCTATCCAGAGGAAAGTAGCCGTGGAG R: CTCCACGGCTACTTTCCTCTGGATAGCGGACATGCTTCGTGTTGTTCTA
**C419G**	F: AACAACACGAAGCATGTCCAATGGCCAGAGGAAAG R: CTTTCCTCTGGCCATTGGACATGCTTCGTGTTGTT
**C419P**	F: AGAACAACACGAAGCATGTCCAATCCCCAGAGGAAAGTAGC R: GCTACTTTCCTCTGGGGATTGGACATGCTTCGTGTTGTTCT
**V425M**	F: GCCAGAGGAAAGTAGCCATGGAGGCTGTCATTCAG R: CTGAATGACAGCCTCCATGGCTACTTTCCTCTGGC
**A427P/I429P**	F: GAGGAAAGTAGCCGTGGAGCCTGTCCCTCAGAGTCATTTGGATGTC R: GACATCCAAATGACTCTGAGGGACAGGCTCCACGGCTACTTTCCTC

### Transfection

Cells were seeded at a density of 300,000 cells/well in 6 well plates. The next day, the cells were transfected using 0.6136 mg/ml polyethylenimine (PEI, 408727, Sigma-Aldrich) in MES-HEPES buffered saline (50 mM MES, 50 mM HEPES, 75 mM NaCl, adjusted to pH 7.2 with NaOH), using a ratio of 4 µl PEI for 2 μg DNA. DNA and PEI were diluted separately in 100 µl of plain DMEM (without any supplement), combined and after vortexing for 10 sec incubated at room temperature (RT) for 20 min. 200 µl of transfection mix was added to the cells in 2 ml of plain DMEM. After 6 hours, medium containing transfection mix was exchanged for DMEM supplemented with 10% FBS.

### Chemical cell fractionation, whole-cell extract and western blot

Cells were collected, washed twice with ice-cold PBS and resuspended in Sucrose buffer (10 mM Tris·HCl pH 7.5, 20 mM KCl, 250 mM Sucrose, 2.5 mM MgCl _2_, 10 mM Benzamidine Hydrochloride and P2714 protease inhibitor cocktail from Sigma-Aldrich). Triton X-100 was added to a final concentration of 0.2%, and cells were vortexed three times for 10 seconds, followed by centrifugation (500 g, 4°C, 5 min) to separate the cytoplasmic fraction from the nuclei pellet. Nuclei were extracted for 30 min on ice in NETN150 buffer (50 mM Tris·HCl pH 8.0, 150 mM NaCl, 2 mM EDTA, 0.5% NP-40, 10 mM Benzamidine Hydrochloride and P2714 protease inhibitor cocktail from Sigma-Aldrich). The nuclear fraction and chromatin pellet were separated by centrifugation (1,000 g, 4°C, 5 min). The chromatin pellet was finally solubilised for 1 h on ice using NETN150 buffer supplemented with 2 mM MgCl _2_ and 125 U/ml Benzonase nuclease (71206-3, Novagen). Cytoplasmic, nuclear and chromatin soluble fractions were centrifuged (16,100 g, 4°C, 30 min) to remove cell debris.

For whole-cell extract, the cells were directly lysed in NETN150 buffer supplemented with 2 mM MgCl _2_ and 125 U/ml for 1 h on ice and centrifuged (16,100 g, 4°C, 30 min) to remove cell debris.

For western blot, proteins were separated by SDS-PAGE and transferred to a nitrocellulose membrane. Detection was performed using antibodies against GFP (RRID:AB_439690, G1544, Sigma-Aldrich, 1:2,000 dilution), FLAG (RRID:AB_439702, A8592, Sigma, 1:1,000 dilution), Lamin A (RRID:AB_532254, L1293, Sigma-Aldrich, 1:1,000 dilution), Tubulin (RRID:AB_1904178, 3873, Cell Signaling, 1:5,000 dilution) and histone H3 (RRID:AB_2118462, A300-823A, Bethyl, 1:2,000 dilution).

### Cell survival assay

EUFA1341 cell lines stably expressing FLAG-PALB2 variants were seeded at a density of 7,500 cells/well in 96-well plates and cultured for 24 h before treatment. Cells were then grown in the presence of 0–100 nM camptothecin (208925, Calbiochem) for 4 days. Cell proliferation was measured using WST-1 reagent (05015944001, Roche Applied Science), as previously described ^24^. 4-day survival is expressed as the percentage of live cells compared to the untreated (no drug) sample. Two technical replicates were performed for each of three experiments. The dose-response curves were fitted to the data pool and the IC50 values calculated using Prism 7 (RRID:SCR_002798, GraphPad Software).

### Immunofluorescence staining and automatic nuclear foci quantification

In 12-well plates, approximately 100,000 EUFA1341 cells complemented with FLAG-PALB2 variants were seeded onto glass coverslips pre-coated, 5 min at room temperature, with 1 µg/ml PEI (408727, Sigma-Aldrich) in plain DMEM. The next day, the growth medium was refreshed with medium supplemented with 10 nM CPT in DMSO or the equivalent volume of vehicle. After 17h of incubation (37°C, 5% CO _2_), the cells were washed twice with 1x PBS and fixed with 4% formaldehyde (Pierce, 28908) in 1x PBS (15 min, RT). The cells were immediately incubated with 125 mM Glycine (Sigma-Aldrich, G7126) in 1x PBS (5 min, RT), to quench the formaldehyde and terminate the cross-linking reaction. The cells were washed twice with 1x PBS and permeabilised with 0.5% Triton X-100 in 1x PBS (5 min, RT). After two additional PBS washes, the coverslips were blocked with antibody dilution buffer (ADB: 1% BSA, 0.2% Cold Water Fish Skin Gelatin, 0.05% Triton X-100 in 1x PBS; 30 min, RT). Rabbit anti-Rad51 (7946 ^28^, 1:1000 dilution) and mouse anti-γH2A.X (RRID:AB_309864, 05-636, Millipore, 1:500 dilution) primary antibodies were applied (2h, RT). After incubation with primary antibodies, the coverslips were washed twice with 0.05% Triton X-100 in 1x PBS (PBS-T) and once with ADB (5 min on an orbital shaker, RT). Following incubation (1h at RT) with anti-mouse Alexa Fluor 555 (RRID:AB_2535846, A-21425, Invitrogen, 1:400 dilution) and anti-rabbit Alexa Fluor 488 (RRID:AB_2534114, A-11070, Invitrogen, 1:400 dilution) secondary antibodies, the coverslips were washed three times with PBS-T (5 min on an orbital shaker, RT). The coverslips were finally air-dried at RT and mounted on glass slides with ProLong Gold antifade reagent with DAPI (P36935, Invitrogen).

Images acquired on an Olympus Fluoview FV1000 confocal laser-scanning microscope, using fixed parameters, were converted to RGB TIFF format using the Fiji (RRID:SCR_002285) distribution of ImageJ ^29^. RAD51 and γH2AX nuclear foci were automatically quantified using the FoCo algorithm ^30^. Noteworthy, filters were applied for the minimum radius of nuclei (15 pixels, blue), the minimum radius of foci (γH2A.X: 3 pixels, red; RAD51: 2 pixels, green) and the minimum intensity of foci (γH2A.X: 0.4, red; RAD51: 0.24, green). The number of foci of the first 180 cells scored was analysed using Prism 7 (RRID:SCR_002798, GraphPad Software).

### Statistics and quantitative analysis

For experiments reproduced at least three times in this study, statistical significance was determined using the indicated test (Student’s *t* test or extra sum-of-squares *f* test). Data were analysed using Excel 2011 for Mac (Microsoft Software) and Prism 7 (RRID:SCR_002798, GraphPad Software).

For the quantitative analysis of PALB2 level in the chromatin fraction, western blots imaged with film were scanned on a Canon 8800F scanner using backlight illumination. For each protein of interest, an area of fixed size was used to calculate the band intensity in the Fiji (RRID:SCR_002285) distribution of ImageJ ^29^. The intensity of the PALB2 band was normalised against the intensity of the corresponding Lamin A band. The level of PALB2 in the chromatin fraction was then normalised against the protein level in the whole-cell extract and finally expressed as a percentage of the normalised wild-type PALB2 chromatin level.

## Results

**Figure 1.**
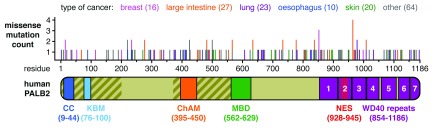
Schematic representation of PALB2 domains and the missense substitutions referenced in the COSMIC database. PALB2 diagram depicting the assigned domains as follows, CC: coiled-coil domain, KBM: KEAP1-binding motif, ChAM: chromatin association motif, MBD: MRG15-binding domain and WD40 repeats 1 to 7. Oblique and vertical striped areas respectively indicate DNA binding regions and nuclear export signal (NES). The histogram indicates the distribution and location of 160 missense substitutions referenced in the COSMIC database. The type of cancer is colour-coded according to the legend and the number of cases indicated in between brackets.

As part of our on-going efforts to characterise the function and regulation of PALB2 chromatin association, we investigate the link between perturbations of PALB2 chromatin association and cancer. To this end, we interrogated the COSMIC (Catalogue Of Somatic Mutations In Cancer) database ^31^ to retrieve missense substitutions within the ChAM (human PALB2 residues 395-450). At the time of writing, the COSMIC database references 263 mutations from 30698 tested samples (0.86%), including 160 missense substitutions spanning the whole length of PALB2 ( Figure 1). When we initiated this analysis, four distinct missense substitutions were identified in the ChAM: P405T, V410G, T413S, and V425M.

### Cancer-associated missense substitutions in the ChAM are predicted to be deleterious

**Figure 2.**
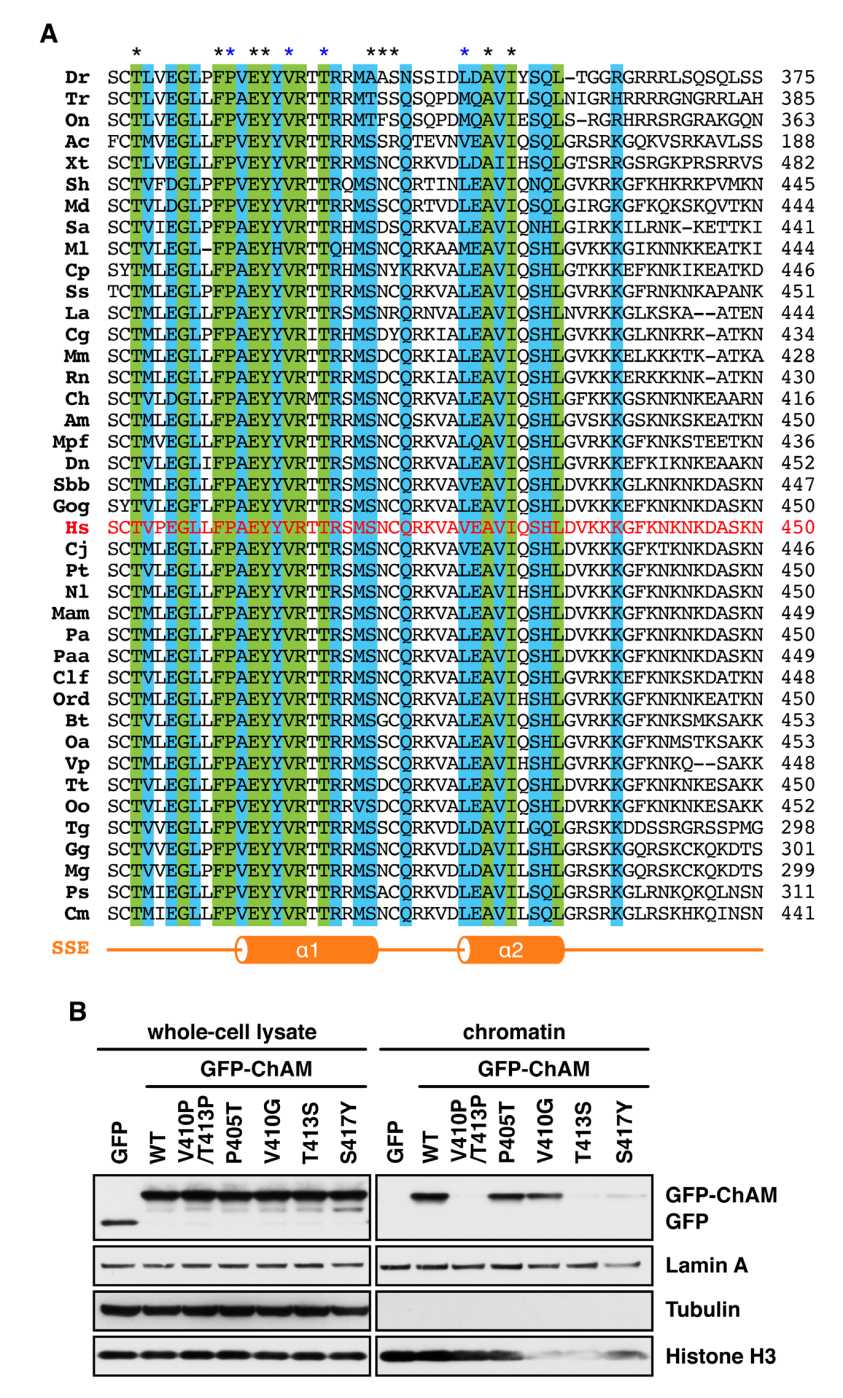
Cancer-associated missense substitutions impair ChAM function. ( **A**) Sequence alignment of the ChAM of 40 species. Red letters highlight the Human PALB2 sequence; green and blue boxes respectively show residues identical in all species analysed and residues with conservative substitutions, across the whole alignment. Asterisks indicate ChAM residues mutated in this study (blue asterisks highlight residues with missense substitutions referenced in the COSMIC database). Secondary structure elements (SSE) of the ChAM as predicted by Jpred4; α: alpha helix. ( **B**) Chromatin association of GFP-ChAM wild-type (WT) and GFP-ChAM bearing the indicated missense substitutions examined by western blot, following transient expression in HEK293T cells. Tubulin, lamin A and histone H3 are markers for extraction of cytoplasmic, nuclear and chromatin proteins, respectively.

The primary amino acid sequence of the ChAM is highly distinctive and does not present any similarities with other known nucleosome-binding domains. With only predicted secondary structure available ( Figure 2A), the amount of information on the structural elements and the overall fold of the domain is also limited. Hence, we first examined the degree of conservation of these residues by aligning the protein sequences of 40 orthologues of PALB2 ( Figure 2A). P405, V410 and T413, are identical in all 40 orthologues of PALB2 analysed, while V425 is a conserved residue with some variation. We next used PolyPhen-2 ^32^ and SIFT ^33^ prediction tools to obtain an initial assessment of the potentially deleterious effect of these amino acid substitutions ( Table 2). Both algorithms predicted P405T, V410G, and T413S substitutions to be deleterious, while the V425M substitution was predicted to be deleterious only by SIFT.

**Table 2.** ChAM missense mutations, deleterious effect prediction and chromatin localisation. Red and green boxes respectively highlight ChAM missense mutations resulting in impaired chromatin association and those showing no obvious defects, as an arbitrary way to discriminate between potentially harmful and benign mutations.

Residue change(s)	PolyPhen score ^a^	SIFT score ^b^	Predicted deleterious	Chromatin localisation	Accession number	Cancer type [reference]
**T397S**	1.000	0.03	yes	Wild type	dbSNP ^d^ [rs367578415]	Breast ^8^
**F404A** **/P405A**	1.000 1.000	0.00 0.00	yes	Wild type	This study	–
**F404L**	1.000	0.00	yes	Wild type	dbSNP ^d^ [rs148921082]	–
**P405T**	1.000	0.00	yes	Wild type	COSMIC ^c^ [COSM4941093]	Liver
**E407A** **/Y408A**	1.000 1.000	0.00 0.00	yes	Wild type	This study	–
**Y408H**	1.000	0.00	yes	Wild type	dbSNP ^d^ [rs515726064]	Breast ^34^
**V410G**	1.000	0.00	yes	Wild type	COSMIC ^c^ [COSM1172313]	Oesophagus ^36^
**N418A** **/C419I**	0.820 0.937	0.08 0.01	yes	Wild type	25	–
**C419P**	0.968	0.02	yes	Wild type	25	–
**C419G**	0.763	0.06	no	Wild type	25	–
**V425M**	0.023	0.01	yes	Wild type	COSMIC ^c^ [COSM1286951]	Autonomic ganglia ^37^
**V410P** **/T413P**	1.000 1.000	0.00 0.01	yes	Impaired	25	–
**T413S**	1.000	0.04	yes	Impaired	COSMIC ^c^ [COSM326058]	Lung ^38^
**S417Y**	1.000	0.00	yes	Impaired	dbSNP ^d^ [rs45510998]	Breast ^8, 10, 17, 39– 42^
**A427P** **/I429P**	0.999 1.000	0.00 0.00	yes	Impaired	25	–

^a^Variants with PolyPhen-2 scores ≥ 0.850 are predicted to be deleterious (highlighted in red). ^b^Variants with SIFT scores ≤ 0.05 are predicted to be deleterious (highlighted in red). ^c^COSMIC: Catalogue Of Somatic Mutations In Cancer database ^31^. ^d^dbSNP: NCBI database of genetic variation ^35^.

### T413S and S417Y missense substitutions hinder PALB2 chromatin association

To directly assess their impact on PALB2 chromatin association, we used site-directed mutagenesis to generate GFP-ChAM variants bearing these missense substitutions and transiently expressed them in HEK293T cells. We additionally tested the effect of three published germline missense substitutions (T397S, Y408H, and S417Y) ^8, 10, 34^ and one variant of unknown significance referenced in the NCBI database of genetic variation (F404L) ^35^. These all affect highly conserved residues and are predicted to be deleterious by the PolyPhen-2 and SIFT algorithms ( Figure 2A and Table 2). Surprisingly, most missense substitutions affecting highly conserved residues retained a wild-type level of chromatin association ( Figure 2B and Table 2). To further confirm this observation, we tested two additional variants with predicted deleterious mutations at two consecutive conserved residues (F404A/P405A and E407A/Y408A) and again were not able to detect any effect on ChAM-mediated chromatin association ( Figure 2A and Table 2). In sharp contrast, we found that T413S and S417Y substitutions dramatically reduced the chromatin association of the GFP-ChAM peptide ( Figure 2B), to a comparable extent as our previously reported ChAM null mutant, where V410 and T413 residues are replaced by two helix-disrupting prolines ^25^.

**Figure 3.**
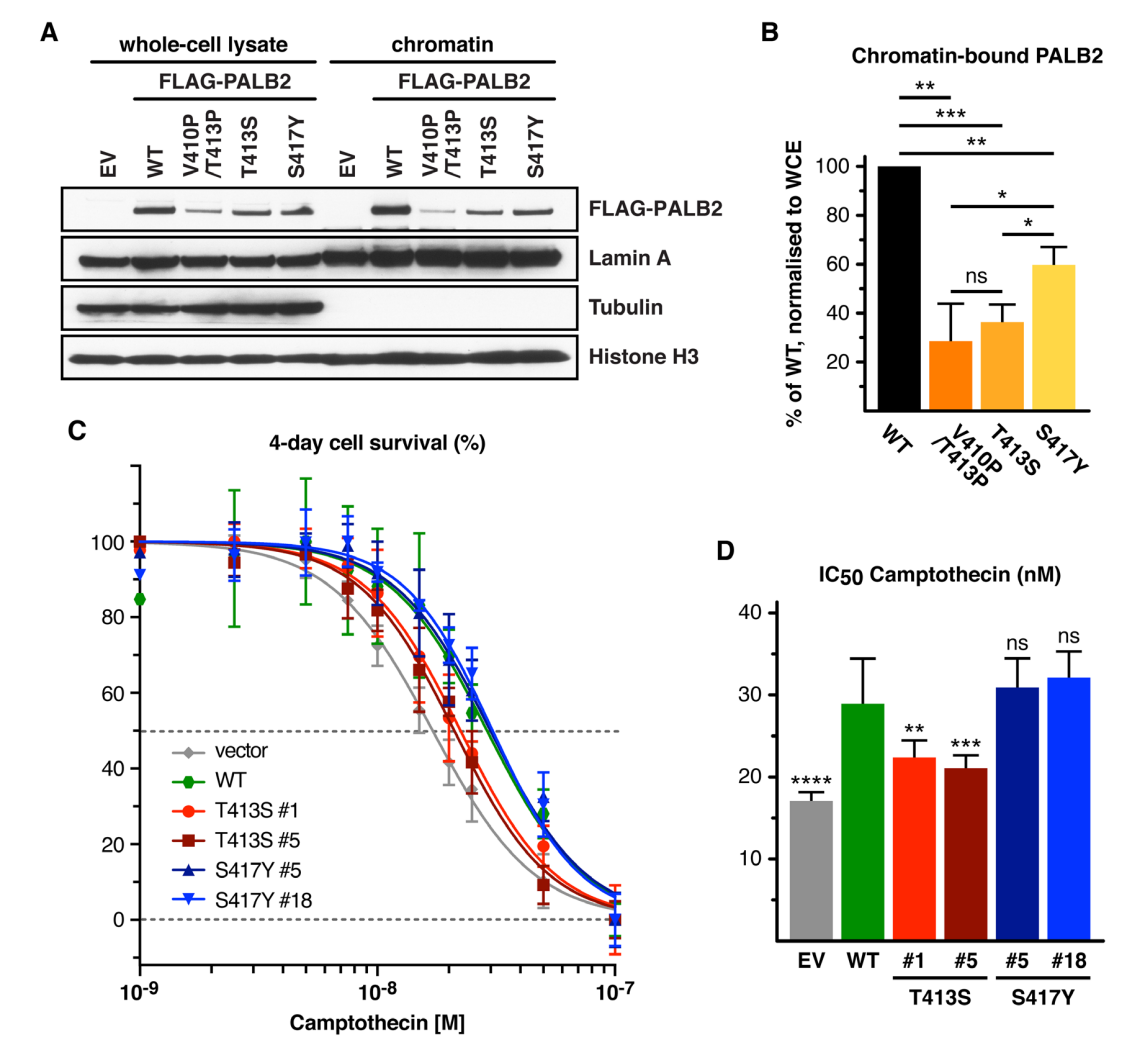
PALB2 with small cell lung cancer T413S mutation behaves as a ChAM null variant. ( **A**) FLAG-PALB2 variants accumulation in the chromatin fraction of stably transfected EUFA1341 cells, as examined by western blot. ( **B**) The level of each FLAG-PALB2 variant in the chromatin fraction was quantified and, following normalisation against their respective level in whole-cell extract and loading control, expressed as % of the wild type (WT). Mean values ±SD (n=4). Asterisks indicate *P* values for two-tailed paired Student’s *t* test with *<0.05, **<0.01 and ***<0.001, ns: non-significant. ( **C**) CPT survival curves fitted from a WST-1 cell proliferation assay comparing EUFA1341 cells complemented with FLAG-PALB2 WT and the indicated variants. Mean values ±SD (n=3). ( **D**) IC50 values for CPT treatment in EUFA1341 complemented cells. n=3, with two technical replicates. Error bars indicate 95% CI. Asterisks indicate the *P* values for the extra sum-of-squares *f* test with **<0.01, ***<0.001 and ****<0.0001, ns: non-significant. EV: empty vector.

We next set to examine the effect of T413S and S417Y missense substitutions on full-length PALB2 chromatin association. Since PALB2 can form homo-oligomers ^19^, we used PALB2-deficient EUFA1341 cells to stably express FLAG-PALB2 variants, while avoiding interference from endogenous PALB2. Interestingly, despite our best efforts to find cell lines with similar level of expression of the FLAG-PALB2 variants analysed, all missense variants displayed a lower level of expression compared to wild type FLAG-PALB2. This observation raises the possibility that ChAM missense substitutions partly destabilise the PALB2 protein and could contribute to the observed PALB2 haploinsufficiency for tumour suppression ^35, 36^. Nevertheless, we observed hindered chromatin association of FLAG-PALB2 with T413S or S417Y missense substitutions, when compared to wild-type FLAG-PALB2 ( Figure 3A). Compared to our analysis of GFP-ChAM variants, full-length PALB2 variants, including the ChAM null mutant, only result in a partial loss of chromatin association. This is somewhat expected since PALB2 chromatin association is mediated not only by the ChAM but also through MRG15 binding ^24– 26^. However, when we quantified the level of chromatin-associated FLAG-PALB2, we found that only the T413S missense substitution recapitulated the phenotype of our ChAM null mutant ( Figure 3B), with 35% of the wild-type level of chromatin association. On the other hand, the S417Y missense substitution exhibited an intermediate phenotype, with a chromatin association level between that of the wild-type and T413S FLAG-PALB2 variants. These results support that the cancer-associated T413S missense mutation is distinctive in causing an overall reduction of PALB2 chromatin association.

### T413S missense substitution impairs PALB2 function in genotoxic drug resistance

The topoisomerase I inhibitor camptothecin (CPT) is a broad-spectrum anticancer drug, and we recently demonstrated that loss of ChAM function sensitises cells to CPT treatment ^25^. To further assess the adverse effects of T413S and S417Y missense substitutions, we examined the ability of these PALB2 variants to support cellular resistance to genotoxic stress in response to chronic exposure to CPT. EUFA1341 cells complemented with wild-type and S417Y FLAG-PALB2 displayed comparable levels of survival ( Figure 3C and D), with IC50 values of ~ 30 nM CPT and overlapping growth inhibition curves. In stark contrast, T413S FLAG-PALB2 was unable to fully rescue the CPT sensitivity of EUFA1341 PALB2-deficient cells, which is reminiscent of our previously described ChAM null variant ^25^.

**Figure 4.**
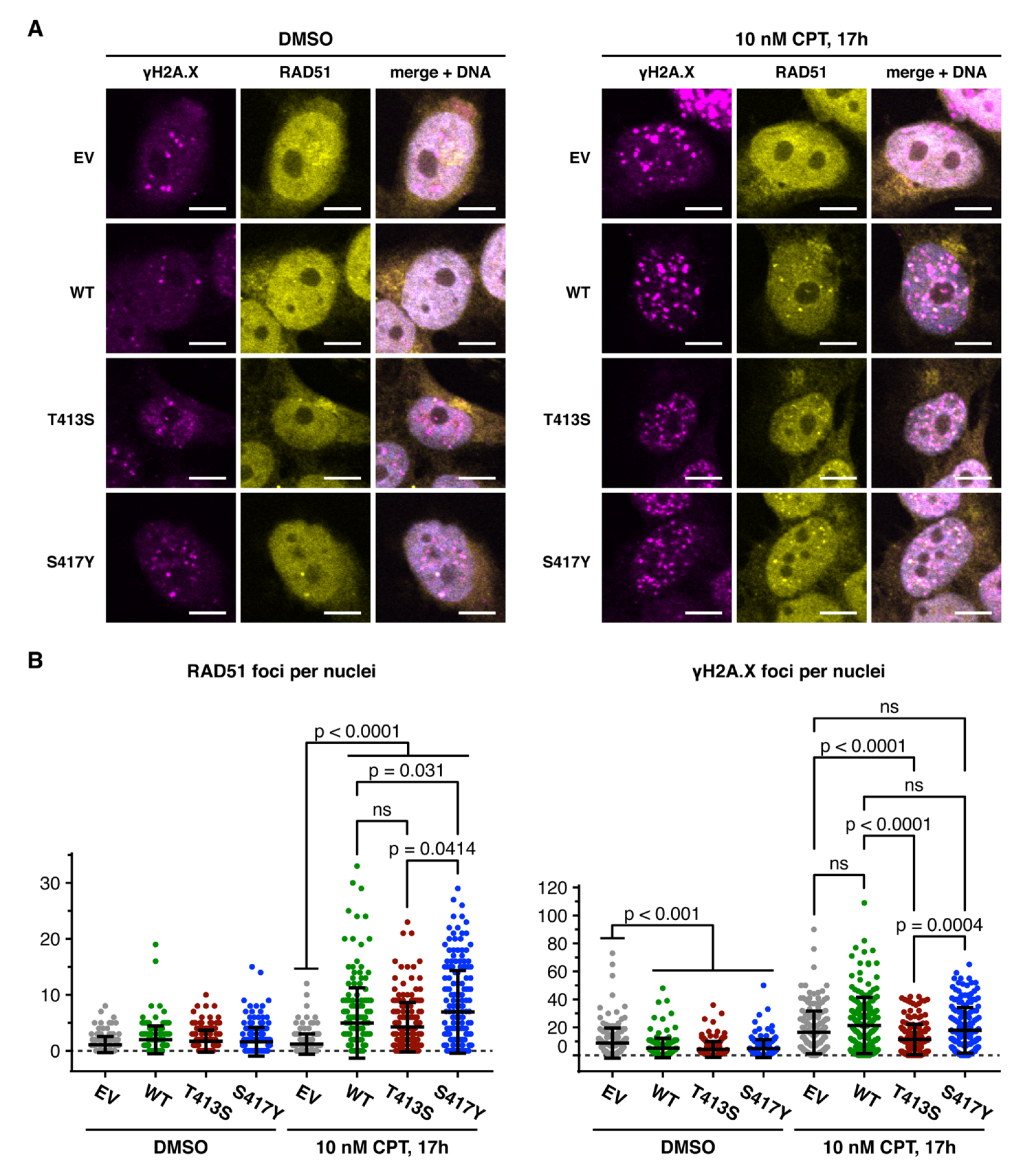
PALB2 T413S missense variant supports RAD51 but not γH2A.X nuclear foci formation. ( **A**) Representative pictures, from one experiment, of EUFA1341 cells complemented with FLAG-PALB2 variants and stained for RAD51 (yellow) and γH2A.X (purple) nuclear foci following 17 h treatment with DMSO (left) or 10 nM CPT (right). ( **B**) Automatic quantification of γH2AX (left) and RAD51 (right) nuclear foci using the FoCo algorithm. Cells were treated for 17h with DMSO or 10 nM CPT, as indicated. Dots represent individual cells (n=180), and bars mean values ±SD. *P* values indicate the statistical significance for the two-tailed Mann-Whitney *U* test. EV: empty vector.

We next sought to gain insights into the mechanism underlying the increased CPT-sensitivity of the PALB2 T413S mutant. Since PALB2 is commonly described to play an essential role in HDR by recruiting BRCA2 in complex with RAD51, we scored RAD51 nuclear foci as a proxy to estimate the efficiency of HDR. As expected, CPT-induced RAD51 nuclear foci formation was severely impaired in EUFA1341 cells ( Figure 4). To our surprise, however, re-expression of wild-type, T413S, and S417Y FLAG-PALB2 in EUFA1341 cells restored similar levels of RAD51 nuclear foci formation upon CPT treatment ( Figure 4B). Although a moderate decrease and increase of the number of RAD51 foci are noticeable in the T413S and S417Y PALB2 variants, respectively, these variations are statistically not significant and are unlikely to explain the differences observed in our CPT survival assay ( Figure 3D and Figure 4B). To further verify the efficacy of the DNA stress response in each cell line, we examined the formation of S139-phosphorylated H2A.X (γH2A.X) nuclear foci, a marker of the activation of the DNA damage response ( Figure 4). In vehicle-treated cells, the expression of wild-type, T413S, and S417Y FLAG-PALB2 equally suppressed the formation of γH2A.X nuclear foci arising from spontaneous DNA lesions in EUFA1341 cells. Intriguingly, however, CPT treatment induced a notably less efficient γH2A.X nuclear foci formation in EUFA1341 cells complemented with FLAG-PALB2 T413S ( Figure 4B). Collectively, these findings demonstrate T413S as a ChAM defective variant of PALB2, yet point to an unexpected mechanism where DNA stress signalling, rather than RAD51 recruitment to DNA lesions, is altered.

## Discussion

Since the discovery of *PALB2* in 2006, numerous studies had addressed how the development of various cancers could be linked with *PALB2* mutations. For the ~50 protein-truncating mutations classified as cancer risk variants, there is abundant evidence that BRCA2 binding is lost since these variants recapitulate many features of BRCA2 loss of function ^5, 10, 16, 18^. Conversely, many missense mutations remain as variants of uncertain significance (VUSs), even though the likely impact of PALB2 missense VUSs hindering BRCA2 or BRCA1 interaction is foreseeable based on the importance of the BRCA1-PALB2-BRCA2 complex in the maintenance of genome stability ^4– 7, 22, 23^. There is also an indication that PALB2 is haploinsufficient for tumour suppression, since almost all PALB2 truncating mutations conferring a risk for breast/ovarian cancer are monoallelic ^43, 44^. This notion was further supported by the recent identification of a hypomorphic allele of PALB2, with an in-frame deletion of residues 839-862 ^21^. This variant can support PALB2 function when exogenously expressed in U2OS cells, but not in patient cells where it is expressed at a low level.

In the present study, we provide additional evidence that impaired PALB2 chromatin association may be linked with cancer development. We demonstrate that two cancer-associated missense mutations in the ChAM of PALB2 hinder its chromatin association ( Figure 3A and B). The serine to tyrosine substitution at position 417 leads to a partial reduction of ChAM-mediated PALB2 chromatin association, without affecting the cellular resistance to CPT ( Figure 3A and Figure 4). However, seven independent studies reported PALB2 S417Y as a germline mutation in both cancer patients (mainly breast cancer) and healthy individuals ^8, 10, 17, 39– 42^, making this variant the most frequent of the subset we analysed. Although further clinical evidence is required, our observations suggest that PALB2 S417Y may be a low-penetrance genetic variant associated with low cancer risk or a neutral variant.

Surprisingly, the second variant, a conservative threonine to serine substitution at position 413 has a more deleterious effect ( Figure 3C and D), recapitulating the phenotypes of our previously described ChAM null allele ^25^. To start dissecting the molecular mechanism underlying the CPT-survival defect associated with PALB2 T413S missense variant, we analysed RAD51 and γH2A.X nuclear foci formation in cells challenged with CPT. Remarkably, we found that PALB2 T413S supported a nearly normal level of RAD51 nuclear foci formation, but a reduced level of γH2A.X nuclear foci formation ( Figure 4B). This observation is somewhat puzzling since most studies report a robust correlation between RAD51 and γH2A.X nuclear foci formation. Nonetheless, the exact nature, meaning and significance of DNA repair proteins nuclear foci remain unclear ^45^, and the RAD51 nuclear foci observed in PALB2 T413S expressing cells do not necessarily reflect regular HDR events. It is conceivable that, through its interaction with BRCA1, PALB2 T413S can initiate RAD51 recruitment, but for as-yet-unknown reasons, fail to promote HDR. Notably, BRCA1, in complex with BARD1, can promote the proteasome-mediated degradation of H2A.X ^46, 47^, and in this way, supports the attenuation of the γH2A.X-mediated damage signalling following the completion of DNA damage repair ^46^. In the case of PALB2 T413S, BRCA1 might be aberrantly trapped at sites of DNA damage due to unproductive HDR events, which then leads to the premature attenuation of the γH2A.X-mediated response ( Figure 4). While additional studies will be required to define a potential role of ChAM in HDR, our observations support the notion that PALB2 T413S perturbs DNA stress signalling, resulting in a decreased cellular resistance to CPT treatment ( Figure 3C and D). Of note, the T413S PALB2 variant change a residue potentially targeted by protein kinases. Although no direct evidence indicates that kinases phosphorylate this particular residue *in vivo*, it is tempting to speculate that changes in ChAM phosphorylation, rather than its primary amino-acid sequence, could be deleterious, affecting productive DNA stress signalling and repair.

**Figure 5.**
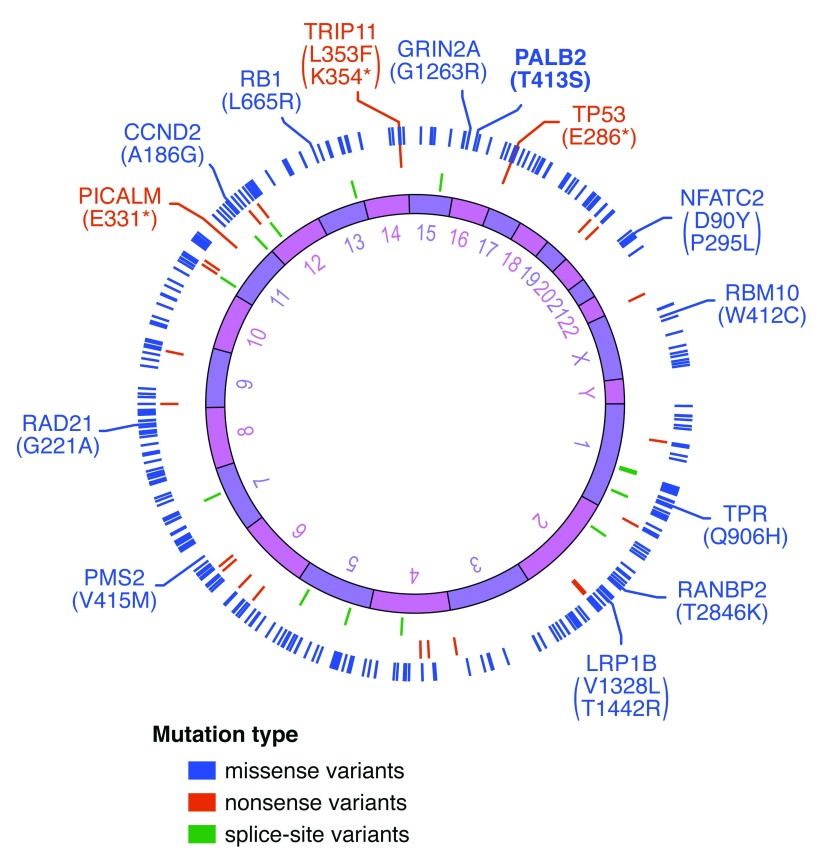
Mutations landscape for the small cell lung cancer case bearing the PALB2 T413S variant. Circos plot depicting the mutations identified by whole exome screening of the SCLC sample 134427 carrying the PALB2 T413S variant. The middle ring shows the chromosomal position, with the missense, nonsense and splice-site mutations arranged on the outside (colour-coded according to the legend). The genes from the COSMIC Cancer Genes Census, for which mutations have been causally implicated in cancer, are annotated.

The PALB2 T413S variant is, however, scarce and reported as a somatic mutation in a single patient with small cell lung cancer (SCLC) ^45^. It is important to note that SCLC is an exceptionally aggressive type of cancer with poor prognosis, and camptothecin (topotecan) is currently the only drug approved by the US Food and Drug Administration to treat relapsed SCLC. While inactivating (nonsense or essential splice-site) mutations of both TP53 and RB1 is associated with a significant fraction of SCLC cases ^38^, whole exome screening of the SCLC patient carrying the PALB2 T413S mutation detected only an inactivating nonsense mutation in TP53 ( Figure 5). Mutations in PTEN, SOX2 and NOTCH1, which are also frequently associated with SCLC, and BRCA1/2 or other known Fanconi anaemia genes were also not detected. Interestingly, the COSMIC database references three additional SCLC cases with rare missense VUSs within PALB2 WD40 repeat domain (Q921H, V978D and R1086G). The link between SCLC and PALB2 mutations is unidentified to date, and although further investigation will be required to assess the pathological consequences of these mutations (namely T413S, Q921H, V978D and R1086G), our findings may have implications for understanding the development of this disease.

Altogether, we screened 15 variants of PALB2 with missense substitutions at 13 different positions of the ChAM, including 11 highly conserved residues ( Figure 2A and Table 2). Given the high level of conservation of the ChAM, it was somewhat surprising to find that most missense substitutions at these residues were well tolerated and did not appear to affect PALB2 chromatin association. Most significantly, our work identifies threonine 413 as a critical residue within the ChAM and implicates its mutation in SCLC. Further investigation of the regulation of PALB2 chromatin association will be essential for the full understanding of its implication for tumorigenesis and to develop new therapeutic strategies.

## Data availability

Raw data for this study are available from OSF: https://doi.org/10.17605/OSF.IO/ZVUK8 ^48^. This includes CPT cell survival data in Microsoft Excel 2011; PALB2 chromatin association quantification data in Microsoft Excel 2011; RAD51 and γH2A.X number of foci per nuclei data in Microsoft Excel 2011; TIFF files of uncropped western blots for Figure 2B and Figure 3A.

Data are available under the terms of the Creative Commons Zero "No rights reserved" data waiver (CC0 1.0 Public domain dedication).
