# Supplementary material for: Perturbation of PALB2 function by the T413S mutation found in small cell lung cancer
Source: Wellcome Open Res. 2018 Jan 18;2:110. Originally published 2017 Nov 14. [Version 2] doi: 10.12688/wellcomeopenres.13113.2 (PMC5721578; doi:10.12688/wellcomeopenres.13113.2)
Supplement: Peer review history

# Peer review: Referee response for version 1

**DOI:** 10.21956/wellcomeopenres.14225.r27998

**Published:** 2017-12-14

**Authors:** Ashok R. Venkitaraman

**Affiliations:** 1 MRC Cancer Cell Unit, University of Cambridge, Cambridge, UK

Mutations affecting the PALB2 gene predispose to cancer. Many cancer-associated mutations truncate the encoded PALB2 protein to varying degrees, disrupting its function in the repair of DNA damage by homologous recombination. This interesting paper builds on previous work from Dr. Esashi’s laboratory to characterize the functional impact of two mis-sense mutations (T413S and S417Y) altering residues in a PALB2 region (chromatin association motif, or ChAM) previously reported to promote nucleosome interaction.

 The authors find that while ChAM fragments bearing the T413S or S417Y mutations exhibit sharply reduced chromatin interaction, full-length PALB2 mutants containing T413S or S417Y still retained a significant proportion of their chromatin-interacting ability, with PALB2 T413S being more severely affected. Consistent with this finding, PALB2 T413S when expressed in PALB2-mutant EUFA1341 cells sensitises them to the topoisomerase inhibitor camptothecin (CPT). In addition, PALB2 T213S (but not S417Y) reduces gamma-H2AX formation after DNA damage, although it has little effect on damage-induced RAD51 foci. The authors suggest that chromatin-bound PALB2 may have functions in the signalling of damage-induced stress distinct from roles in RAD51 recruitment, and that disruption of such functions may account for cancer promotion by mutations affecting the ChAM.

 The experiment shown in Figure 3A/B is not easy to interpret. Figure 3A shows that FLAG-tagged PALB2 T413S and S417Y are expressed in significantly lower amounts than the FLAG-PALB2 wild-type protein in whole-cell extracts, and their degree of chromatin association appears proportional to the level of expression (as reflected in Figure 3B). Therefore, is the reduction in mutant PALB2 protein expression – most severe for the functionally compromised T413S mutant – responsible for both the reduced chromatin association and the observed cellular defects?

 In this regard, the authors’ finding that GFP-tagged T413S or S417Y ChAM fragments exhibit little detectable chromatin association (Figure 2B), whereas full-length PALB2 mutants containing the same mutations retain significant chromatin binding (Figure 3A) is intriguingly unexplained.

 The authors make the interesting point that the PALB2 S417Y variant, which has little functional effect in their studies apart from reduced protein expression, has been found in the germline of cancer patients as well as healthy individuals. While it is possible,  as the authors suggest, that this variant represents a low-penetrance cancer gene, it may also represent a neutral variant (particularly if it affects only one of the two PALB2 germline alleles).

 Finally, the authors raise the interesting possibility that the PALB2 T413S mutant, identified as a somatic mutation in a single patient with lung cancer, may promote carcinogenesis and resistance to drugs like CPT. However, it was not clear from the accessible sequencing data whether the PALB2 T413S mutation affected one or both PALB2 gene copies in this patient’s sample. Should a functional wild-type copy of PALB2 be retained in this patient’s tumour, experiments showing that the PALB2 T413S mutant can act as a “dominant negative” even when wild-type PALB2 protein is present would strengthen the authors’ conjectures regarding pathogenic impact. This issue should ideally be explained and discussed, in case there are potential limitations to the authors’ conclusions regarding the clinical significance of their findings.

I have read this submission. I believe that I have an appropriate level of expertise to confirm that it is of an acceptable scientific standard.

# Response

**Published:** 2018-01-12

**Authors:** Fumiko Esashi

**Affiliations:** University of Oxford, UK

**The experiment shown in Figure 3A/B is not easy to interpret. Figure 3A shows that FLAG-tagged PALB2 T413S and S417Y are expressed in significantly lower amounts than the FLAG-PALB2 wild-type protein in whole-cell extracts, and their degree of chromatin association appears proportional to the level of expression (as reflected in Figure 3B). Therefore, is the reduction in mutant PALB2 protein expression – most severe for the functionally compromised T413S mutant – responsible for both the reduced chromatin association and the observed cellular defects?**

We appreciate this comment. As described in the figure legend, we normalised the signal intensities of PALB2 variants against loading control (Lamin A) in the chromatin fraction, which was then normalised against the respective PALB2/Lamin A ratio in the whole-cell extract; in this way, we ensured that the quantification reflects the proficiency of PALB2 chromatin association. Nonetheless, we agree that the expression levels of PALB2 mutant proteins are lower compared to wild-type PALB2, and now discuss the possible link with PALB2 haploinsufficiency for tumour suppression.

**In this regard, the authors’ finding that GFP-tagged T413S or S417Y ChAM fragments exhibit little detectable chromatin association (Figure 2B), whereas full-length PALB2 mutants containing the same mutations retain significant chromatin binding (Figure 3A) is intriguingly unexplained.**

We appreciate this comment. The partial chromatin association of full-length PALB2 variants is expected, as wild-type PALB2 chromatin binding is mediated through two mechanisms: direct interaction between ChAM and nucleosomes (Bleuyard et al., 2012 EMBO Rep; this study), and the stable interaction with MRG15, which binds to trimethylated histone H3 at K36 (Bleuyard et al., 2017 PNAS). When both mechanisms are perturbed, full-length PALB2 fails to associate with chromatin. This point is now clarified in the text.

**The authors make the interesting point that the PALB2 S417Y variant, which has little functional effect in their studies apart from reduced protein expression, has been found in the germline of cancer patients as well as healthy individuals. While it is possible, as the authors suggest, that this variant represents a low-penetrance cancer gene, it may also represent a neutral variant (particularly if it affects only one of the two PALB2 germline alleles).**

We agree with this comment and have amended the text accordingly.

**Finally, the authors raise the interesting possibility that the PALB2 T413S mutant, identified as a somatic mutation in a single patient with lung cancer, may promote carcinogenesis and resistance to drugs like CPT. However, it was not clear from the accessible sequencing data whether the PALB2 T413S mutation affected one or both PALB2 gene copies in this patient’s sample. Should a functional wild-type copy of PALB2 be retained in this patient’s tumour, experiments showing that the PALB2 T413S mutant can act as a “dominant negative” even when wild-type PALB2 protein is present would strengthen the authors’ conjectures regarding pathogenic impact. This issue should ideally be explained and discussed, in case there are potential limitations to the authors’ conclusions regarding the clinical significance of their findings.**

This is a valid point, and it was not possible to obtain the information about the second PALB2 allele in this patient. The PALB2 T413S mutant shows increased sensitivity, rather than resistance, to camptothecin treatment, and to date, we have no evidence that PALB2 T413S acts as a ‘dominant negative’. The pathological consequence of this single point mutation is as yet unknown, and we have amended the text accordingly.

# Peer review: Referee response for version 1

**DOI:** 10.21956/wellcomeopenres.14225.r27997

**Published:** 2017-12-06

**Authors:** Kevin Hiom

**Affiliations:** 1 Division of Cancer Research, Medical Research Institute, Jacqui Wood Cancer Centre, Ninewells Hospital and Medical School, Dundee, UK

This manuscript reports the characterization of cancer-associated mutations in the chromatin associated motif of PALB2 identified through mining of the COSMIC database and analysis of published literature. The authors identified two mutations T413S and S417Y that exhibited defects in chromatin binding when expressed in HEK293T cells with only the former defective in chromatin binding in EUFA13141. Interestingly the T413S mutation has been found in a single case of small cell lung cancer. While expression of the PALB2 T413S mutant in EUFA13141 sensitized these cells to treatment with camptothecin, localization of RAD51 to sites of DNA damage was near normal. Unexpectedly, gH2AX foci in these cells was reduced compared to cells expressing wild type PALB2. The authors conclude that PALB2 that is impaired for chromatin binding is compromised for DNA damage signalling while seemingly proficient in the recruitment of downstream repair proteins. They propose that chromatin binding is required for DNA damage signalling, which might impact on the ability of cancer cells to respond to treatment with the therapeutic agent camptothecin.

 This study is a well presented and interesting study describing a functional requirement for the chromatin association motif of PALB2. It demonstrates clearly that mutations in the ChAM that reduce retention of PALB2 on chromatin have an impact on the DNA damage response. What is less clear is why? At the current time the investigation of ChAM mutants on the DDR is limited. It is clear that expression of T413S causes sensitization to camptothecin but this was not reflected in decreased RAD51 recruitment or increased gH2AX foci. However these assays were performed at a single time point and the data does not reflect the kinetics of repair. Is RAD51 recruitment affected over time? Does the decrease in gH2AX foci reflect fewer collapsed replication forks or increased rate of dsb repair (albeit possibly a harmful form of repair leading to decreased survival)? These issues are not addressed and this might explain the difficulty in reconciling a defect in DDR signalling with apparently normal recruitment of downstream repair factors but impaired survival. Given that PALB2 has, through BRCA1 and BRCA2, been linked to homologous recombination repair it might have been useful to quantify levels of HR and investigate sensitivity to Parp inhibitors.

 Minor points-

1. The western blot for levels of histone H3 seems irregular
2. Conclusions suggesting the potential importance of ChAM mutations in PALB for the pathology of SCLC are overstated given the single instance of this mutation in the disease.

I have read this submission. I believe that I have an appropriate level of expertise to confirm that it is of an acceptable scientific standard.

# Response

**Published:** 2018-01-12

**Authors:** Fumiko Esashi

**Affiliations:** University of Oxford, UK

**This study is a well presented and interesting study describing a functional requirement for the chromatin association motif of PALB2. It demonstrates clearly that mutations in the ChAM that reduce retention of PALB2 on chromatin have an impact on the DNA damage response. What is less clear is why? At the current time the investigation of ChAM mutants on the DDR is limited. It is clear that expression of T413S causes sensitization to camptothecin but this was not reflected in decreased RAD51 recruitment or increased gH2AX foci. However these assays were performed at a single time point and the data does not reflect the kinetics of repair. Is RAD51 recruitment affected over time? Does the decrease in gH2AX foci reflect fewer collapsed replication forks or increased rate of dsb repair (albeit possibly a harmful form of repair leading to decreased survival)? These issues are not addressed and this might explain the difficulty in reconciling a defect in DDR signalling with apparently normal recruitment of downstream repair factors but impaired survival. Given that PALB2 has, through BRCA1 and BRCA2, been linked to homologous recombination repair it might have been useful to quantify levels of HR and investigate sensitivity to Parp inhibitors.**

We are grateful for these very insightful comments and suggestions. We totally agree that the molecular and biological functions of the ChAM domain remain unknown, despite the clear phenotypes supporting its role in the DNA damage response. We are currently tackling the question of why the ChAM defect leads to increased sensitivity to camptothecin using various experimental approaches, including those suggested by the referee. We hope to be able to provide clearer answers in our follow-up studies.

**Minor points-**

**1. The western blot for levels of histone H3 seems irregular**

This is a blotting artefact due to the bleaching of the histone H3 signal, which was used a marker of the chromatin-associated fraction. As described in the methods, however, we used Lamin A, whose molecular weight is closer to GFP-ChAM, and not histone H3 for normalisation. This does not affect our conclusion.

**2. Conclusions suggesting the potential importance of ChAM mutations in PALB for the pathology of SCLC are overstated given the single instance of this mutation in the disease.**

We appreciate this comment and have amended the text accordingly.

# Peer review: Referee response for version 1

**DOI:** 10.21956/wellcomeopenres.14225.r27996

**Published:** 2017-11-27

**Authors:** Grant W. Brown, Tajinder Ubhi

**Affiliations:** 1 Department of Biochemistry, Donnelly Centre, University of Toronto, Toronto, ON, Canada

This manuscript describes the identification of cancer-associated missense mutations within the chromatin-association motif of the tumor suppressor gene *PALB2*. Although the role of *PALB2* missense mutations in disrupting its protein interactions with complex members BRCA1 and BRCA2 is well-established, the role of missense mutations in perturbing PALB2 chromatin-binding function and its contribution to tumorigenesis is unknown. These potentially high-impact mutations were identified by mining the public cancer mutation database, COSMIC, and the literature. The fifteen identified missense mutations in the chromatin-association motif were introduced into HEK293T cells, and two mutations (T413S and S417Y) were found to have impaired chromatin binding, one of which is found to be mutated in a single case of SCLC (T413S). These two missense mutations were further introduced into full-length *PALB2 *and expressed in *PALB2-*deficient EUFA13141 cells, where only the T413S mutation was found to perturb chromatin binding. PALB2 T413S-expressing cells are more sensitized to camptothecin treatment, and display reduced levels of g-H2AX foci compared to cells expressing wild-type PALB2 and PALB2 S417Y while maintaining comparable levels of Rad51 foci. This suggests failure to efficiently activate DNA damage repair signalling while maintaining downstream effector recruitment. Overall, the authors propose a novel mechanism through which *PALB2 *missense mutations could promote tumorigenesis by impairing its chromatin-binding ability, albeit found in a very limited number of tumours to date.

**Comments:**

1. The immunoblot in Figure 2B does not show consistent levels of histone H3 in the chromatin extracts. Although likely an artefact, a better example would strengthen the finding of decreased chromatin binding of GFP-ChAM fragments harbouring T413S and S417Y mutations.
2. It appears that all of the mutants cause a reduction in steady-state PALB2 levels. I appreciate that this could be an artefact of stable cell line selection, but probably worth a comment nonetheless, given the possible relationship between PALB2 haploinsufficiency and disease.
3. In Figure 4B, shouldn’t comparisons be made between cells expressing WT PALB2 and the ChAM-mutant PALB2 proteins, in addition to the PALB2-deficient empty vector? The comparison shown, with EV, suggests that T413S is interfering with the action of another effector protein, since it is more defective than cells lacking PALB2. Assuming that the difference between WT and T413S is statistically supported, the data also indicate that T413S is defective relative to WT.
4. In the Discussion, the statement “While the exact role of ChAM in HDR remains to be elucidated” seems premature, given there is no evidence yet of an HDR defect in the mutants studied.
5. “The link between SCLC and PALB2 mutations is unidentified to date, and our findings may have significant implications for understanding the development of this disease.” I would omit ‘significant’ given that the mutation seems to be a part of the minority in SCLC and its functional consequences are as of yet undefined.
6. The PALB2 ChAM-null variant response to camptothecin would be a good comparison to include in Figures 3C and 3D.
7. Scale bars should be added to the micrographs in Figure 4A.
8. A couple of typos. Results section two header reads S417S instead of S417Y, and ‘cell lines’ should be singular in the following sentence: “To further verify the efficacy of the DNA stress response in each cell lines, we examined….”.

We have read this submission. We believe that we have an appropriate level of expertise to confirm that it is of an acceptable scientific standard.

# Response

**Published:** 2018-01-12

**Authors:** Fumiko Esashi

**Affiliations:** University of Oxford, UK

**Comments:**

**1. The immunoblot in Figure 2B does not show consistent levels of histone H3 in the chromatin extracts. Although likely an artefact, a better example would strengthen the finding of decreased chromatin binding of GFP-ChAM fragments harbouring T413S and S417Y mutations.**  

This is indeed a blotting artefact due to the bleaching of the histone H3 signal in the enhanced chemiluminescence (ECL) reaction. Histone H3 was used as a marker of the chromatin-associated fraction, but Lamin A, whose molecular weight is closer to GFP-ChAM, was used for normalisation as described in the methods. The bleached histone H3 signal therefore does not affect our conclusion. Building on the severe phenotype detectable with the GFP-ChAM harbouring T413S and S417Y, we moved on to in-depth analyses of these mutations in the full-length PALB2 context (Figure 3).

**2. It appears that all of the mutants cause a reduction in steady-state PALB2 levels. I appreciate that this could be an artefact of stable cell line selection, but probably worth a comment nonetheless, given the possible relationship between PALB2 haploinsufficiency and disease. **  

We appreciate this comment. Indeed, we generated several stable clones expressing these PALB2 mutants, but were unable to obtain clonal lines expressing equivalent levels. In the revised manuscript, we discuss the level of expression of FLAG-PALB2 variants and the possible link with PALB2 haploinsufficiency for tumour suppression.

**3. In Figure 4B, shouldn’t comparisons be made between cells expressing WT PALB2 and the ChAM-mutant PALB2 proteins, in addition to the PALB2-deficient empty vector? The comparison shown, with EV, suggests that T413S is interfering with the action of another effector protein, since it is more defective than cells lacking PALB2. Assuming that the difference between WT and T413S is statistically supported, the data also indicate that T413S is defective relative to WT.**  

We agree with this comment and have now included the statistical data comparing wild-type PALB2 to T413S or S417Y variants, and the statistical data comparing the T413S and S417Y variants.

**4. In the Discussion, the statement “While the exact role of ChAM in HDR remains to be elucidated” seems premature, given there is no evidence yet of an HDR defect in the mutants studied.**  

We have now amended this statement as the following: “While additional studies will be required to define a potential role of ChAM in HDR”.

**5. “The link between SCLC and PALB2 mutations is unidentified to date, and our findings may have significant implications for understanding the development of this disease.” I would omit ‘significant’ given that the mutation seems to be a part of the minority in SCLC and its functional consequences are as of yet undefined.**  

We appreciate this comment and have amended the text accordingly.

**6. The PALB2 ChAM-null variant response to camptothecin would be a good comparison to include in Figures 3C and 3D.**  

We included the ChAM-null variant (V410P/T413P) in our CPT survival analysis, but unfortunately, had trouble adjusting the seeding for this particular cell line, as reflected by the high WST-1 assay OD reading (50-100% higher than other cell lines) from raw CPT survival data (available on OSF). The elevated cell density for the ChAM-null mutant affected the efficiency of CPT killing, especially at high CPT doses, and, for this reason, we did not include these data in the manuscript.

**7. Scale bars should be added to the micrographs in Figure 4A.**  

Scale bars have now been added to Figure 4A and the legend amended accordingly.

**8. A couple of typos. Results section two header reads S417S instead of S417Y, and ‘cell lines’ should be singular in the following sentence: “To further verify the efficacy of the DNA stress response in each cell lines, we examined….”.**  

We thank the referee for detecting these typos and have corrected them in the revised manuscript.
